# Equitable access to digital higher education for students with disabilities in South Africa

**DOI:** 10.4102/ajod.v14i0.1525

**Published:** 2025-02-28

**Authors:** Johannes N. Zongozzi, Sindile A. Ngubane

**Affiliations:** 1 Institute for Open Distance Learning, College of Education, University of South Africa, Pretoria, South Africa

**Keywords:** higher education, accessible education, digital learning, students with disabilities, South Africa

## Abstract

**Background:**

The growing reliance on digital learning in South Africa, partly because of the emergence of the coronavirus disease 2019 (COVID-19) and 4IR technologies, risks excluding students with disabilities (SwDs) if measures to adequately support them are not in place.

**Objectives:**

The study aims to identify gaps in knowledge, policies, practices and resources, which could impede the full engagement of SwDs. This article utilises the conceptual framework for inclusive digital learning, which comprises three categories of concepts related to inclusive digital learning: (1) defining attributes, (2) antecedents (necessary conditions) and (3) consequences (results). The framework is applied to synthesise the literature, determine the framework’s efficacy, feasibility, and suitability, and demonstrate its value and utility in the actual implementation of inclusive and high-quality higher education for SwDs in South Africa during this era of digital learning.

**Method:**

This study reviewed 22 articles (2020–2023) on disabilities, higher education, and digital learning identified through Google Scholar using Boolean operators.

**Results:**

The study reveals significant gaps in South African higher education research on institutional policies related to digital access, capacity development, and disability inclusion in teaching and curriculum design.

**Conclusion:**

The challenges facing SwDs and the existing research gaps imply that most higher education institutions lack the theoretical knowledge, policies, resources, infrastructure and staff capacity to support SwDs.

**Contribution:**

This study exposes gaps in the literature and recommends further research on higher education policies and to establish the potential for policy reform to better support SwDs in the current era of digital learning.

## Introduction

### Background

The global phenomenon of systematically excluding people with disabilities (PwDs) from all aspects of the economy has elicited responses from various stakeholders (Ongolo 2018; Sipuka 2019; Sipuka & Ngubane 2022). Several global and regional treaties, such as the United Nations Convention for the Rights of Persons with Disabilities (UNCRPD), along with national legislation, highlight the importance of taking proactive measures to address these inequalities, which are expected to worsen because of the increasing dependence on Fourth Industrial Revolution (4IR) technologies. The requirement for PwDs to have access to quality education as a prerequisite for economic participation is explicitly stated in international agreements such as the UNCRPD and the UN Convention on Human Rights (United Nations 2008).

In response to this urgent situation, the Protocol to the African Charter on Human and Peoples’ Rights on the Rights of Persons with Disabilities in Africa (African Union 2018) and the African Union Agenda 2063 (African Union Commission 2015) were adopted. South Africa complies with the aforementioned guidelines through various documents, including the Constitution of the *Republic of South Africa Act 108 of 1996*, the National Plan for Higher Education, the Strategic Policy Framework on Disability for the Post-School Education and Training System (2018), the White Paper 3 on Higher Education Transformation (1997), the *Equity and Prevention of Unfair Discrimination Act 4 of 2000*, and the Education White Paper 6: Special Needs Education (2001).

The shared understanding on matters such as the Sustainable Development Goals (SDGs), United Nations Global Compact (UNGC) principles, and Environmental, Social, and Governance (ESG) has led to several bilateral and multilateral collaborations between South African universities and other institutions. In 2007, the University of South Africa (Unisa) joined the United Nations Global Compact (UNGC). University of South Africa has made a commitment to the Global Compact and has outlined several actions to fulfil this commitment. These actions include incorporating the 10 principles of the Global Compact into university policies and procedures, ensuring that the necessary skills and capacity are in place to carry out their responsibilities, implementing suitable systems and processes to ensure efficient operations, fostering a climate of accountability and responsibility among employees, integrating the 10 UNGC principles into teaching and research, engaging with local and global communities to promote sustainability through partnerships, and supporting and advancing the work of the UNGC, which focuses on principles related to labour, human rights, the environment, and anarchy. Unisa acknowledges that the SDGs may be accomplished by following the 10 principles of the UNGC. The organisation is dedicated to promoting the 2030 Agenda for Sustainable Development, with a particular focus on 12 out of the overall 17 SDGs (University of South Africa 2022).

Hence, the aforementioned international agreements, domestic strategies, and policies are interrelated in facilitating fair and equal opportunities for students with disabilities (SwDs) in South Africa. This is achieved by advocating for inclusiveness, accessibility and the integration of assistive technologies within digital learning platforms among others, thus guaranteeing the inclusion of every student in the ever-changing digital education environment.

Notwithstanding the aforementioned attempts, research on the state of higher education during the coronavirus disease 2019 (COVID-19) pandemic suggests that the sector underwent significant upheaval, necessitating a reconfiguration of its structure. During this period, both faculty and students had to adapt their learning, assessment and student support methods. Consequently, the use of digital learning platforms was necessitated (Tesar 2020). This resulted in a rise in educational inequalities, namely among SwDs, because of the unexpected transition to these digital learning platforms in South African higher education (Ngubane-Mokiwa & Zongozzi 2021). Ngubane-Mokiwa and Zongozzi (2021) found that these modifications resulted in a higher rate of exclusion for SwDs, especially when their assessment had to be rescheduled to the second semester because of student’s lack of preparation for the special online examinations. Students in the study reported that having to take twice as many assessments at the end of the year was an additional cause of stress. Furthermore, as a result of the shutdown of the Post Office during South Africa’s National Lockdown Level 5, some SwDs who lacked the required hardware, software and bandwidth were unable to access supplementary materials in alternative formats such as Braille or large print. Consequently, many students expressed their discontent with their inability to get the assistive devices they often borrowed from the library and receive through Post Office.

Prior studies have identified several difficulties or barriers to accessing quality higher education for SwDs in the South African digital learning context. These include inaccessible learning materials, a lack of awareness and identification of SwDs, insufficient capacity and support for lecturers (Chidindi 2012; Zongozzi 2020), and poor implementation of relevant policies (Mutanga, Manyonga & Ngubane-Mokiwa 2018; Odhav 2009; Zongozzi 2020). This provides a rationale to synthesise the literature on equitable access to higher education for SwDs during the digital learning age in South Africa to gain a better understanding of the problem.

### Research problem

The context described earlier provides an insight into the potential for increased marginalisation of SwDs in South Africa because of the increasing dependence on digital learning systems. Because of the endorsement of international and regional treaties discussed earlier, as well as the national policy and legislative framework, this will constitute a breach of SwD’s right to high-quality education and consequently create an inequitable chance for participation in the economic sector.

Conversely, the South African Education White Paper 3: A Programme for the Transformation of Higher Education (1997) acknowledges the important role of digital learning in terms of expanding access, promoting diversity and enhancing efficiency. This is attributed to the diverse contexts in which learning occurs in this mode, encompassing a range of platforms, individualised pacing, utilisation of various media, and implementation of diverse teaching and learning methodologies. Therefore, this method of teaching and learning can help the government to achieve its goal of promoting and enhancing social justice. Through the use of digital learning platforms, institutions can have the capacity to accommodate a larger number of students simultaneously, ensuring fairness and equal opportunities (Zongozzi 2020). This phenomenon can be attributed to the fact that an increasing number of individuals are depending on online courses, as technological advancements continue to progress (Lee & Choi 2011). Nevertheless, the aforementioned policy’s goals of transforming the higher education sector through expanding access, promoting diversity and enhancing efficiency is defeated by the protracted systematic exclusion of SwDs where the inevitable use of digital learning platforms without adequate research and policies to guide the needed knowledge, skills and resources to fully engage SwDs undermines their right to high-quality education.

### Objective of the research

The objective of this study is to conduct a review and synthesis of the current literature on SwDs in digital learning environments in South Africa in order to identify gaps in knowledge, policies, practices and resources that impede the full engagement of SwDs. It is hoped that the logical conclusions of this study may serve as policy recommendations and strategies that promote full inclusion and guarantee fair educational opportunities for all students. This article utilises the conceptual framework for inclusive digital learning to synthesise the literature, determine the framework’s efficacy, feasibility, and suitability, and demonstrate its value and utility in the actual implementation of inclusive and high-quality higher education for SwDs in South Africa during this era of digital learning. From a realist perspective, it is expected that if the relationship between the elements of the framework for inclusive digital learning in the transformation of higher education in South Africa is properly configured (Zongozzi 2022), it will provide a clear explanation of the factors that enable it. Otherwise, it will highlight the obstacles towards the aforementioned necessity.

#### Conceptual framework for inclusive digital learning

**FIGURE 1:**
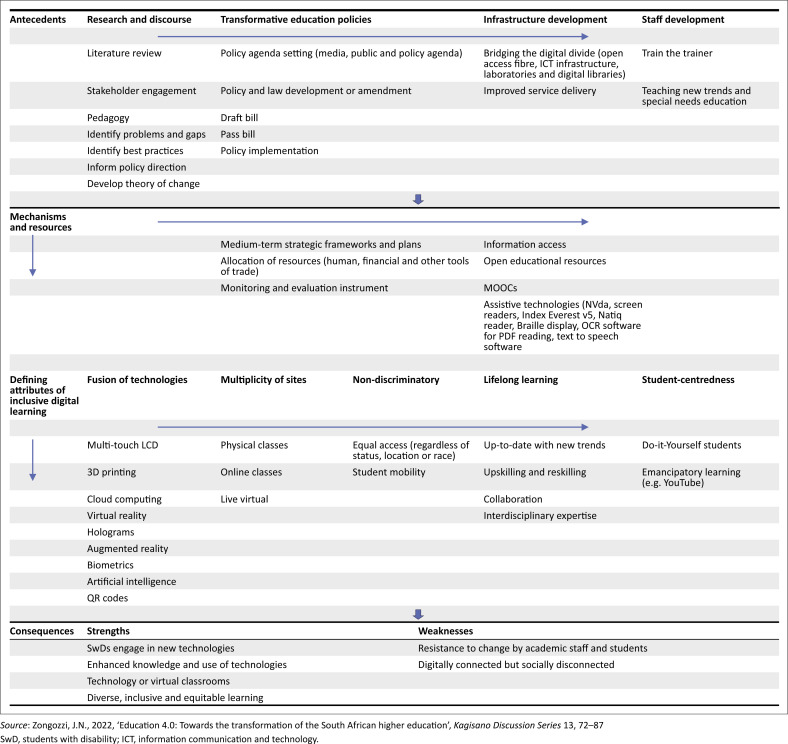
Conceptual framework for inclusive digital learning.

Zongozzi (2022) initially formulated the conceptual framework for inclusive higher education in the era of the 4IR as presented in Figure 1, by employing a concept analysis technique proposed by Walker and Avant (2014). The analysis commences with identifying the concept in question, specifically the facilitation of inclusive digital learning for SwDs within the framework of transformational higher education. The analysis entails examining the several uses of the concept, establishing its defining attributes and identifying typical model and borderline instances related to the concept. Antecedents, which refer to the preceding events and elements necessary for the manifestation of a concept, are also a vital component of this framework. The consequences, or the end results that arise from the concept, are likewise a crucial facet of the framework (Walker & Avant 2014).

The framework for this research as illustrated in Figure 1 comprises three categories of concepts related to inclusive digital learning: (1) defining attributes, (2) antecedents (necessary conditions) and (3) consequences (results). Defining attributes refer to the features that are typically associated with a concept and the phenomena it reflects (Walker & Avant 2014). The defining attributes for inclusive digital learning, as identified through a preliminary review of academic research and policy materials, encompass the fusion of technologies (Butt et al. 2020; Goh & Aris 2015; Naidoo & Singh-Pillay 2020), the multiplicity of sites (Ally & Wark 2020; Hussin 2018; Scepanović 2019), the provision of non-discriminatory educational opportunities (*Constitution of the Republic of South Africa Act 108 of 1996* [Republic of South Africa 1996]; *Promotion of Equality and Prevention of Unfair Discrimination Act 4 of 2000* [Republic of South Africa 2000]; Department of Education 1997; Department of Education 2001), the promotion of lifelong learning (Fomunyam 2016; Yusuf, Walters & Sailin 2020; Ally & Wark 2020) and the implementation of student-centred teaching and learning that fosters empowerment (Ally & Wark 2020; Reaves 2019; Waghid, Waghid & Waghid 2019).

The framework asserts that in order for the defining attributes of inclusive digital learning to be apparent, specific antecedents (necessary conditions) should precede them (Walker & Avant 2014). These encompass research and discourse (Khan 2016; Steglitz et al. 2015), policies that bring about significant changes (Butt et al. 2020; National Policy Development Framework 2020 [Republic of South Africa 2020]), the construction of necessary facilities (Ravhudzulo 2019; Zongozzi 2022) and the enhancement of skills and abilities of university staff (Ally & Wark 2020; Naidoo & Singh-Pillay 2020; Yusuf et al. 2020). Furthermore, the consequences of a concept pertain to the specific events or happenings that occur as a direct outcome of the manifestation of the concept (Walker & Avant 2014). Therefore, the outcomes obtained from the configured elements of the framework can be seen as a direct consequence of the concept of digital learning, given that all the defining attributes of the concept appear to be present.

The primary objective of this conceptual framework is to provide guidance for the creation of inclusive higher education institutions that enable SwDs to actively engage in the digital learning environment of the 4IR (Desai & Johnson 2013). The framework proposes an intricate interdependence among its three primary components and variables. The ultimate result of efforts to offer comprehensive digital education for SwDs depends on the interplay between the antecedents and the attributes that define inclusive digital learning.

Zongozzi’s (2022) framework proposes that engaging in research and discourse, which leads to the adoption of transformative education policies, the development of ICT-related infrastructure and the capacity building of academic and support staff in higher education, will inevitably unlock the necessary strategic plans, human resources and financial resources.

## Research methods and design

This article presents the results of a literature review study that was conducted to uncover the fundamental concepts that underpin this research (Peters et al. 2015). This facilitated the identification and illustration of the knowledge gaps in the subject being investigated, as well as the absence of research conducted within the framework of inclusive education in South Africa, specifically during the current era of digital transformation (Munn et al. 2018). The review provided a comprehensive summary of the current body of knowledge (Peters et al. 2015), facilitated the synthesis and distribution of research findings, and offered suggestions for future study (Tricco et al. 2016).

The purpose of this study was to synthesise the literature, assess the effectiveness, practicality, and suitability of a pre-existing framework, and demonstrate its value and utility in the real-world implementation of inclusive digital learning for SwDs in South Africa, particularly in the context of the digital learning era. The components of the framework were used as a priori areas to focus our examination of the secondary literature to achieve this purpose. Given that the pertinent material is found in journal articles, it was imperative to initially seek guidance from the university’s librarian regarding the optimal course of action. Consequently, a discussion was held on Microsoft Teams in June 2023, during which she provided guidance to the researcher throughout the entire process.

The search was conducted using Google Scholar, a web search engine that indexes scholarly literature in many publishing formats and fields. Google Scholar provides free access to full text or metadata of these articles. We ensured this by accessing the platform via our institutional log in, thereby allowing access to full texts of articles in databases to which the institution subscribes. The key phrases and search string listed hereafter were used to extract information from Google Scholar using Boolean Operators: *Disability* AND *South Africa* AND *Open Distance eLearning* OR *Open Distance Learning* OR *Digital learning* OR *online learning*. The original search yielded a total of 36 200 articles. The author utilised the custom range option offered by the search platform, which enabled us to limit our search results to articles published between 2020 and 2023. This is a period where COVID-19 began, leading to an increased reliance on digital learning. This study included only articles that were classified as scholarly peer-reviewed journal articles by Google Scholar filters and were accessible in their full-text format. A total of 114 articles remained following this process.

Importantly, the aforementioned remaining articles underwent assessment to determine their credibility and relevance to the issue being studied. This entailed taking into account the publication date to ensure that the work is within the specified study period (2020–2023) and specifically focused on South African higher education. Furthermore, this method involved evaluating if the article covers the particular elements of special education, inclusivity and the use of digital technology for learning, as these concepts are central to this study. This process resulted in further exclusion of 92 articles because they were considered irrelevant to the topics of accessibility for SwDs and the use of digital learning in the higher education setting in South Africa.

After eliminating all irrelevant publications based on the given criterion, a total of 22 articles were considered suitable for analysis in this study. Table 1 presents a list of articles reviewed (*N* = 22):

**TABLE 1:** Articles reviewed (*N* = 22).

Article number	Article’s full description
1	Aluko, F.K. & Mampane, M.R., 2022, ‘Students with disabilities’ access to distance education: A case for transformational leadership within the ambit of Ubuntu’, *International Journal of African Higher Education* 9(1), 94–115. https://doi.org/10.6017/ijahe.v9i1.15237
2	Cebisa, Z.E., 2021, ‘Student support in an open and distance electronic learning (ODeL) context: The experiences of students with disabilities in KwaZulu-Natal’, *The International Journal of Open Distance e_Learning* 7(1), 53–62.
3	Common Wealth of Learning, 2021, ‘Advancing equity and inclusion through open learning’, *Connections: Learning for Sustainable Development* 26(3), 1–16.
4	De Klerk, E.D. & Palmer, J.M., 2022, ‘Technology inclusion for students living with disabilities through collaborative online learning during and beyond COVID-19’, *Perspectives in Education* 40(1), 80–95.
5	Ditlhale, T.W. & Johnson, L.R., 2022, ‘Assistive technologies as an ODeL strategy in promoting support for students with disabilities’, *Technology and Disability* 1(11), 1–15.
6	Dube, N. & Baleni, L., 2022, ‘The experiences of higher education students with disabilities in online learning during the COVID-19 pandemic’, *Journal of Culture and Values in Education* 5(1), 59–77.
7	Kanwar, A., 2021, Research in Open Learning: lessons for a post-COVID world. Virtual presentation.
8	Lyner-Cleophas, M., Apollis, L., Erasmus, I., Willems, M., Poole, L., Minnaar, M. et al., 2021, ‘Disability unit practitioners at Stellenbosch University: COVID-19 pandemic reflections’, *Journal of Student Affairs in Africa* 9(1), 223–234.
9	Makwembere, S., 2022, ‘Systematic review of study designs and methods of research on disability in South African higher education institutions amidst COVID-19 (2020–2021)’, *Journal of Culture and Values in Education* 5(1), 122–143.
10	Manase, N., 2021, ‘Disguised blessings amid COVID-19: Opportunities and challenges for South African University students with learning disabilities’, *Journal of Student Affairs in Africa* 9(1), 107–118.
11	Manase, N., 2023, ‘Self-devised assistive techniques by university students with learning disabilities’, *African Journal of Disability* 12, a1106.
12	Matjila, T.N., 2023, ‘Evaluation of student support services at an open distance and e-learning university: Towards a framework for students who are deaf and hard of hearing’, PhD thesis, Department of Psychology, University of South Africa.
13	Mutongoza, B.H. & Olawale, B.E., 2021, ‘Whitewashed tombs: Emergency online learning through the experiences of students with disabilities at a rural South African University’, paper presented at the International Academic Forum, Barcelona, 2021.
14	Ndlovu, S., 2023, ‘Preparedness and response to COVID-19 disruptions and learning challenges for students with disabilities in South Africa: A systematic review’, *Sustainability* 15(2), 1420.
15	Ngubane-Mokiwa, S.A. & Khoza, S.B., 2021, ‘Using Community of Inquiry (CoI) to facilitate the design of a holistic e-learning experience for students with visual impairments’, *Education Sciences* 11(4), 1–12.
16	Zongozzi, J.N., 2020, Accessible quality higher education for students with disabilities in a South African open distance and e-learning institution: Challenges.
17	Ntombela, S., 2020, ‘Teaching and learning support for students with disabilities: Issues and perspectives in open distance e-learning’, *Turkish Online Journal of Distance Education* 21(3), 18–26.
18	Pete, J. & Soko, J.J., 2020, ‘Preparedness for online learning in the context of COVID-19 in selected Sub-Saharan African countries’, *Asian Journal of Distance Education* 15(2), 37–47.
19	Pitsoane, E.M. & Matjila, T.N., 2021, ‘Experiences of students with visual impairments at an open distance and e-learning university in South Africa: Counselling perspective’, *Journal of Student Affairs in Africa* 9(2), 123–138.
20	Prinsloo, P. & Uleanya, C., 2022, ‘Making the invisible, visible: Disability in South African distance education’, *Distance Education* 43(4), 489–507.
21	Sipuka, O., 2021, ‘Exploring a framework for decolonised disability-inclusive student walk support practices in an open and distance learning institution’, PhD thesis, Department of Health and Disability Studies, University of Cape Town.
22	Ngubane-Mokiwa, S.A. & Zongozzi, J.N., 2021, Exclusion reloaded: The chronicles of COVID-19 on students with disabilities in a South African open distance learning context.

The conceptual framework that was proposed (Zongozzi 2022) was employed as a priori and a lens to facilitate the examination of available studies. The framework was employed in conjunction with content analysis to organise the fragments of text in a systematic manner (Zolnoori et al. 2019). The researcher categorised data into specific framework categories by observing pertinent content in the margin adjacent to the text (Srivastava & Thomson 2009). The data were subsequently organised in an Excel spreadsheet using headings and sub-headings that were consistent with the predetermined inquiries of the study’s conceptual framework. Ultimately, the basic features of the data were collected in order to generate a map and conduct a comprehensive analysis of the entire dataset. This entailed the defining and elucidation of terms, the illustration of the variety and type of phenomena present in the data, the development of typologies, the establishment of connections and the development of ‘bottom-up’ explanations for these. It also entailed the recommendation of intervention and practice strategies as required (Parkinson et al. 2016).

### Ethical considerations

This article does not contain any studies involving humans or animals performed by any of the authors.

## Results

### Research studies on the antecedents of inclusive digital learning

#### Research and discourse

The bounded review of South African articles published from 2020 to 2023 reveals significant advancements in the discourse surrounding the accessibility of education and SwDs within the current era of digital revolution. Most of the reviewed articles focused mainly on themes such as assistive technologies or technology inclusion (De Klerk & Palmer 2022; Ditlhale & Johnson 2022; Manase 2023), student support (Cebisa 2021; Matjila 2023; Ntombela 2020; Sipuka 2021), experiences, challenges, opportunities, lessons and/or solutions (Dube & Baleni 2022; Kanwar 2021; Manase 2021; Mutongoza & Olawale 2021; Ndlovu 2023; Pitsoane & Matjila 2021; Prinsloo & Uleanya 2022; Singh-Pillay & Khumalo 2021; Zongozzi 2020), higher education institution’s preparedness (Pete & Soko 2020), methodology and theories (Makwembere 2022; Ngubane-Mokiwa & Khoza 2021), equity, inclusion and/or inequalities (Common Wealth of Learning 2021; Ngubane-Mokiwa & Zongozzi 2021), leadership (Aluko & Mampane 2022), and disability units and practitioners (Lyner-Cleophas et al. 2021).

The emergence of the COVID-19 pandemic and its impact on digital learning have contributed to the deserved popularity of the aforementioned study themes. The broader aspects of research in distance education, such as access and equity (Zawacki-Richter 2009), have arguably gained greater significance and urgency. However, in order to ensure that education is accessible and fair for SwDs and those from disadvantaged backgrounds, it would have been appropriate to focus on researching macro-level issues such as higher education policy related to remote education (Zawacki-Richter 2009). Policy research considerations play a crucial role in the adoption of transformative education policies that are essential for inclusive digital learning. Such policies would minimise the negative effects of the digital divide, provide assistive learning technologies and enhance the capacity of higher education staff, among other factors (Butt et al. 2020; Chidindi 2012; Lourie 2017; Zongozzi 2020). Hence, the absence of research on education policies undermines most efforts, as seen from the perspective of the proposed conceptual framework for inclusive digital learning.

#### Transformative education policies

In 2022, Dr Blade Nzimande, the Minister of Higher Education Science and Innovation, released a call for public comment on the Draft Policy for the Recognition of South African Higher Education Institutional Types. This announcement was published in the Government Gazette No. 47205, Notice 2359 on Monday, 08 August 2022. The draft policy aims to establish a higher education system that can effectively cater to the diverse backgrounds, needs, interests and abilities of future students. Its goal is to enable students to reach their full potential and contribute the knowledge, insight, skill and capability required for the development and reconstruction of our country. This aligns with the objectives outlined in the White Paper on Higher Education and Post School Education and Training (Republic of South Africa 2022). Notwithstanding this endeavour, research on policy developments on education and transformation remains crucial, as effective policies necessitate a strong theoretical foundation. Implementing policies and strategies that are flawed in their theoretical design may not be feasible (Khan 2016).

From the reviewed articles, Manase (2023) argued that the university under study had not implemented its own disability policy at the point of conducting her study. The researcher was provided with a draft policy that lacked explicit definitions of both assistive technology and disability. Instead, it provided a clear definition and explanation of disabilities within the framework of the medical model of disability (Manase 2023). This can worsen the difficulties that students with invisible disabilities (including learning disabilities) encounter in accessing resources, particularly when their impairment is closely tied to a decline in physical abilities, the author argues.

To promote equitable access to digital education for SwDs, Pete and Soko (2020) advocated for internet providers to offer subsidised internet bundles and for policymakers to reduce tax levies on internet service providers. This would enable learners and instructors to have affordable and dependable internet connections outside of their educational institution. This finding aligns with Kanwar’s (2021) suggestion for mitigating the increasing disparities. The author contends that governments and organisations should make efforts to design policies that specifically cater to the needs of marginalised groups such as women, girls, people in distant regions and persons with disabilities. By doing so, they would effectively serve the entire population.

Pitsoane and Matjila (2021) contended that the existing policies and implementation plans fail to effectively address the actual circumstances, primarily because of a lack of coordination in handling disability matters and delayed referral of students to counselling services. They recommended that disability issues be given higher priority and integrated into broader strategic plans of the university to expedite their implementation. The effort will involve training ICT staff in various computer software programs required to assist visually impaired students, creating alternative assessments for both formative and summative evaluations, establishing a job readiness intervention programme for graduates to enhance their financial independence and contribute to the university’s employment equity goals, and finally, prioritising the disability unit’s role in coordinating all disability-related matters. Perhaps these endeavours would effectively tackle Zongozzi’s (2020) concern over policy shortcomings in addressing the implementation of disability-related matters in teaching and curriculum design, as well as the training of staff to handle such issues. In addition, it can also tackle issues related to the insufficient adoption of open-access policies, which therefore leads to a lack of capacity among academic staff in promoting inclusive learning (Ngubane-Mokiwa & Khoza 2021).

#### Infrastructure development

The articles also addressed concerns related to the development of digital learning infrastructure to accommodate SwDs. Dube and Baleni (2022) argue that the shift to digital learning is challenging and has an impact on a broader range of individuals, not simply those with disabilities. Network and Wi-Fi issues are reported to have a widespread impact on online learning in South Africa. This is particularly hard because of the relatively high cost of mobile data and Wi-Fi, as well as the presence of network coverage problems in certain areas of the country. Therefore, the difficulties encountered by SwDs vary significantly based on the specific nature of their disabilities and geographical locations. This declaration from the Commonwealth of Learning (COL) (2021) confirms that the lack of targeted support, internet connectivity, accessible software and learning materials is likely to increase the disparity for SwDs.

Thus, the aforementioned concerns are extensively discussed in the literature, with Singh-Pillay and Khumalo (2021) and Ngubane-Mokiwa and Zongozzi (2021) also contributing to the discourse. The former emphasises the contextual challenges faced by SwDs in their learning environments, such as the absence of necessary resources such as data, reliable internet connection and assistive devices, which hindered their capacity to engage in online learning. However, the latter discovered structural issues in ICT, which include insufficient availability of hardware and software, as well as aesthetic design that disregards the needs of SwDs. Kanwar (2021) asserts that government policies and funding will play a crucial role in supporting research and development that is aligning with the proposed framework. Ensuring inexpensive and accessible education will heavily rely on the presence of advanced technology infrastructure and reliable connectivity. Universities should enhance their alignment with the needs of society and prioritise research that contributes to sustainable development.

#### Enhancing the skills and capabilities of university staff

The ability of university personnel to support SwDs in the age of the digital revolution also surfaced as a cross-cutting subject in the analysed studies. Zongozzi (2020) contended that SwDs are often labelled as slow, poor performers, incapable or failure when they fail to perform because of a lack of awareness and clear protocols for identifying these students among lecturers and university officials. This lack of identification hinders the provision of appropriate support. Therefore, despite its potential for success, emergency online learning has not been able to provide inclusive education for SwDs. According to a study conducted by Mutongoza and Olawale (2021), it is suggested that educators receive training on how to effectively promote learning using unfamiliar technological interfaces, particularly those used by SwDs for online learning.

The study conducted by Singh-Pillay and Khumalo (2021) highlights the absence of personal interaction between lecturers and SwDs, the importance of actively promoting communication, the necessity of developing particular pedagogical approaches for online teaching, and the obstacles associated with designing inclusive digital learning materials. Generally, face-to-face instruction enables students to participate in many activities, including explaining, directing, scaffolding and questioning. Nevertheless, the authors contend that lecturers frequently transfer their in-person teaching methods directly to their online teaching without making necessary adjustments. Utilising face-to-face material for digital learning does not guarantee accessibility for SwDs. Therefore, it is imperative for lecturers to ensure that the materials they produce are inclusive of those with various disabilities, such as visual, hearing, motor and cognitive impairments. The COVID-19 pandemic revealed the University of Johannesburg’s insufficient ability to serve SwDs, which made academics vulnerable. As a result, training methods were developed to enable them to carry on with their teaching and learning activities. This phenomena necessitated the modification of the academic calendar of the institution and the introduction of online learning tools. It also involved the creation of online assessment tools and obtaining approval from the appropriate academic authorities for any changes to be implemented in the programmes (Ndlovu 2023). Therefore, it is essential that both academics responsible for delivering instruction and administrators and authorities overseeing the online learning platforms receive training. The vulnerability of all stakeholders across the nation makes it necessary to implement a higher education policy that acknowledges the importance of developing staff capability.

Consequently, lecturers do not feel fully equipped to accommodate SwDs because of lack of training and preparedness. Furthermore, it elucidates the rationale behind the perception of SwDs at higher education institutions that their lecturers lack supportiveness. According to a lecturer’s viewpoint, their absence is justified in terms of support because they are not given sufficient training to support SwDs (Zongozzi 2020). Hence, one study suggested that the United Nations Educational, Scientific and Cultural Organization, in conjunction with institutions, should give priority to enhancing the skills and knowledge of lecturers and other users in online education through capacity building (Pete & Soko 2020). According to Ntombela (2020), there is little to no interaction between the disability section, known as the Advocacy and Resource Centre for Students with Disabilities, and academics in a particular university. This is in spite of the fact that the unit is headed by administrators who lack information about the requirements of the curriculum’s mandate to organise advocacy campaigns and advocate for resources.

Considering the aforementioned problems, Manase (2021) proposes the necessity of expanding the range of instructional materials, teaching methods and assessment modalities in order to minimise the disadvantages faced by students with disabilities (SwDs). She suggests incorporating the Universal Design for Learning (UDL) into educational institutions to accommodate all forms of diversity. This can be achieved by designing learning spaces, arranging physical environments, presenting instruction and implementing assessment modes that are accessible and usable by all students, without the need for individualised support, regardless of their specific needs.

### Research studies on the mechanisms and resources for inclusive digital learning

This study corroborates Cebisa’s (2021) assertion that limited resources exacerbate the issue of accessibility for SwDs. One study found that a participant with an intellectual disability expressed the view that disability is a multifaceted phenomena. The participant emphasised that relying just on assistive technologies is insufficient, as he also requires the assistance of a caregiver to help with his daily tasks. This is because his intellectual disability necessitates a longer period of time for studying. In the study conducted by Dube and Baleni (2022), participants did not name any additional assistive technologies provided by the institution, other than user-friendly computers that had enlarged words and symbols specifically designed for visually impaired students. These do not cater for the diverse nature of disabilities nor are they sufficient for the needs of SwDs.

One of the analysed studies found that none of the students were using advanced assistive technology, expressly developed to help with the difficulties caused by learning disabilities (Manase 2023). Although the institution made accommodations for the students during the examinations, which allowed them to not rely on assistive technology, this may not be a long-term solution to ensuring high-quality inclusive digital learning. However, the study found that the inclusive learning methods mentioned earlier included the practice of merely having SwDs take tests and examinations in a smaller and noise-free location, separate from other students. Additional accommodations were made by others to modify the examination settings. These accommodations included providing extra time, assigning scribes to read and write down students’ responses, allowing the use of spell checks and providing isolated cubicles for students who use scribes or who have severe symptoms of a disability.

The COL (2021) argues that the solutions provided thus far have included tailored materials and courses to specifically meet the needs of practitioners who provide support to women, girls, individuals with disabilities, and those active in family and intergenerational literacy. It has also created Open Educational Resources (OER) with the aim of enhancing equal access to education using mobile phones and Aptus devices, in order to reach individuals residing in rural regions. Nevertheless, issues endure in spite of these advancements. Platforms such as SUNLearn, MS Teams and Zoom provide difficulties when using accessible technology such as screen readers and Braille displays. According to Lyner-Cleophas et al. (2021), several students expressed the requirement for further instruction prior to their participation in discussions, ability to access educational resources and completion of assessments. Given the limited academic schedules commonly found in South African higher education, it is noteworthy that students experience a loss of academic time because of the absence of resources and study materials in alternative and easily accessible formats (Pitsoane & Matjila 2021).

Ngubane-Mokiwa and Zongozzi (2021) found that inclusive digital learning for SwDs can be worsened by several factors. These include the socioeconomic disadvantage of most SwDs, the hindrance posed by their disabilities, the prevalence of unfair practices, the exclusion, and inequalities that SwDs face, and the high costs of internet access. If sufficient procedures and resources are not implemented, the aforementioned groups of students are at risk of either failing or dropping out if they do not receive comprehensive help to access online learning and assessment.

### Defining attributes: The current state of inclusive digital education for students with disabilities in South Africa

#### A lack of integration of technology

From the literature examined in the scoping review, it is evident that technologies are not sufficiently incorporated into South African higher education to enable fair and equal access for individuals with disabilities during this era of digital learning. The review indicates that there has been extensive research and discussion on this specific aspect. However, this research has not been adequately connected to studies on education policy (Kanwar 2021; Manase 2023; Pete & Soko 2020). This connection is important for developing transformative education policies that can help bridge the digital divide (Lourie 2017). Research should inform policy efforts to address the concerns that hinder inclusive digital learning for most SwDs. These concerns include their socioeconomically disadvantaged backgrounds, the nature of their disabilities, the prevalence of inequitable practices and the high costs of internet access (Ngubane-Mokiwa & Zongozzi 2021).

#### Inflexibility and immobility of learning

The framework recognises the value of learning from a variety of locations, as it acknowledges the potential benefits that arise from the combination of 4IR technologies. This integration will enable students the mobility and the internationalisation of higher education (Scepanović 2019). The essential characteristic of inclusive digital learning for SwDs cannot be achieved in South Africa because of the existing gaps in research, education policies and the unavailability of equitable technological access. The concerns mentioned in the reviewed articles, such as the absence of assistive technologies and expensive data costs (Ngubane-Mokiwa & Zongozzi 2021), along with the lack of government and institutional efforts to address these issues through inclusive education policies (Kanwar 2021; Pete & Soko 2020), define the discriminatory nature of the current higher education system.

#### Absence of continuous learning

As a result of the shortcomings, the aspiration for lifelong learning to be a common characteristic of inclusive digital learning for individuals with disabilities remains unfulfilled. This presents a potential hazard as it could make certain learners studying in South Africa obsolete for employment in the era of 4IR. The statement is based on the fact that employers in the 21st century are looking for employees who can show innovation, teamwork and entrepreneurial skills. They also need to be able to work well in interdisciplinary teams and incorporate knowledge from different fields into their work. This is because of the fast-paced advancements in technology associated with the 4IR (Fomunyam 2016). Consequently, SwDs are deprived of the chance to stay updated with the latest advancements in the 4IR era because of the absence of continuous learning opportunities. Inclusive digital learning plays a crucial role in enhancing the skills and knowledge of workers at every phase of their professional journey.

#### Discriminatory in nature

Given that higher education is a constitutional obligation that the state must ensure is accessible and available through appropriate means (South Africa 1996), the exorbitant expenses associated with data required for inclusive digital learning, the digital divide and the absence of infrastructure in underprivileged communities inevitably result in the exclusion of individuals from historically marginalised groups. Thus, digital learning might be seen as potentially benefiting those who already have advantages rather than those who do not. This would therefore be considered a breach of constitutional rights and, to some degree, be perceived as discriminatory.

#### Not student-centred

Student-centredness refers to the capacity of students to think in non-traditional ways and actively participate in the process of acquiring knowledge by determining their preferred learning methods. Nevertheless, as a result of the current limitations in technology resources, SwDs in South Africa are forced to depend on traditional methods of learning. This is somewhat counterintuitive as digital learning ought to promote emancipatory learning (Zongozzi 2022).

## Conclusion

The growing dependence of South Africa on digital learning, propelled by the COVID-19 pandemic and 4IR technology, has advantages for several students but may result in the exclusion of SwDs. This study reviewed 22 articles to assess a framework for inclusive digital learning. The findings revealed deficiencies in research, which impede higher education institutions from effectively assisting SwDs because of insufficient theoretical knowledge, resources, policies, infrastructure and staff capabilities. A number of South African universities, such as the Unisa, have joined forces to work on initiatives pertaining to SDGs, UNGC principles and ESG matters. Nevertheless, the lack of research in the current literature regarding the particular topic leads to a decreased focus on issues related to SwDs and digital learning in both government and institutional policies. This study thus recommends the need for additional research on higher education policies and their potential for reform to better support SwDs in the current era of digital learning.
